# Appearances of Premature Atrial and Ventricular Contractions After Switching From Brand Name to Generic Cibenzoline

**DOI:** 10.7759/cureus.41164

**Published:** 2023-06-29

**Authors:** Gaku Oguri, Katsuhito Fujiu, Tsukasa Oshima, Issei Komuro

**Affiliations:** 1 Department of Cardiovascular Medicine, The University of Tokyo, Tokyo, JPN; 2 Department of Advanced Cardiology, The University of Tokyo, Tokyo, JPN

**Keywords:** ventricular arrhythmias, supraventricular arrhythmias, atrial fibrillation, generic drug, cibenzoline

## Abstract

In antiarrhythmic drugs, the therapeutic window is narrower than in other drugs. Brand name and generic drugs do not use the same inactive ingredients and binder substances. We report a 78-year-old male patient who had been treated for asymptomatic atrial fibrillation and atrial/ventricular premature contractions. Brand name cibenzoline had completely suppressed all arrhythmias. When the medication was changed to the generic drug, the patient showed frequent palpitations and arrhythmias. After restarting the brand-name drug, the arrhythmias were completely suppressed again. These results showed clear differences between brand name and generic cibenzoline in a specific patient. This report is the first case to describe the diminished effectiveness of generic oral cibenzoline.

## Introduction

Currently, generic drugs are very common and play an important role in reducing medical expenses. We often prescribe generic drugs, including generic antiarrhythmic drugs, in daily medical practice. However, brand-name drugs and generic drugs are not exactly the same in their inactive ingredients or binder substances. In antiarrhythmic drugs, the treatment window is key for the effect. There is less evidence about generic drugs than about brand-name ones in terms of their effectiveness.

## Case presentation

**Figure 1:**
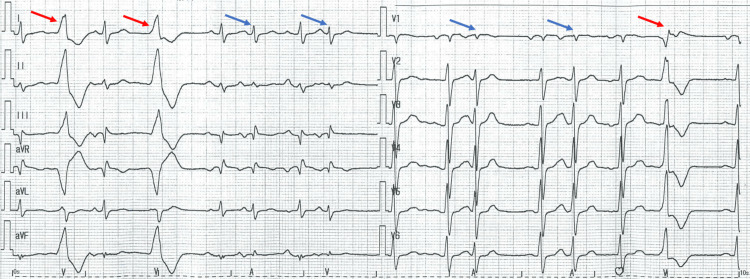
Twelve-lead ECG obtained when the patient presented with complaints of palpitation. ECG showing premature ventricle contractions (red arrows) and atrial premature contractions (blue arrows).

**Figure 2:**
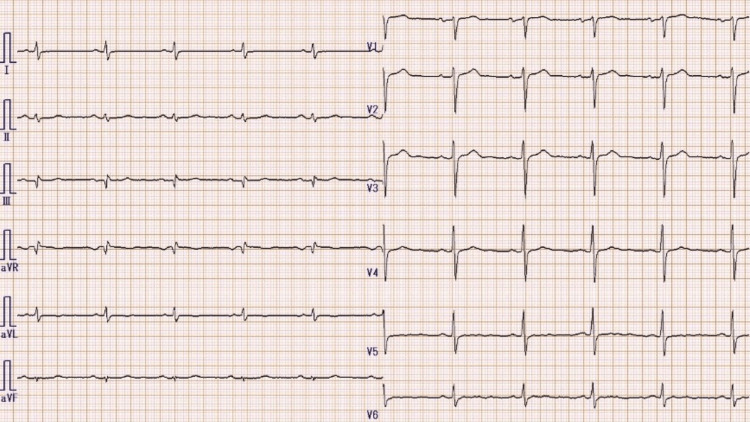
Twelve-lead ECG obtained after one week. At the time, he was taking brand-name drug. ECG showing sinus rhythm without premature ventricle contraction and atrial premature contraction.

We report a case of a 78-year-old male patient who had been treated for paroxysmal atrial fibrillation for 24 years. The patient was prescribed cibenzoline (Cibenol® 100 mg, three times per day (tid)) and recovered a sinus rhythm. He did not present with symptomatic palpitations. Two months prior to this case report, the pharmacist switched the medication from the brand formulation of cibenzoline (Cibenol® 100 mg, tid) to the generic one (cibenzoline succinate 100 mg, tid). The next day, the patient began to present with a different kind of palpitation at the time of paroxysmal atrial fibrillation, and he was admitted to the emergency department. The electrocardiogram demonstrated frequent premature ventricular and atrial contractions (Figure 1). All blood chemical analyses were normal, indicating that the patient did not have hyperkalemia, hypoglycemia, and hyperthyroidism. He was treated for hypertension, hyperthyroidism, and dyslipidemia at the time of this presentation with imidapril (Tanatril®, 10 mg/day), amlodipine (Amlodin OD®, 10 mg/day), levothyroxine sodium (Thyradin S®, 50 μg/day), and atorvastatin (Atorvastatin Tablets®, 5 mg/day). He took these drugs consistently and accurately. We switched cibenzoline from the generic to the brand-name drug. A week later, the ECG showed that premature ventricular contractions were terminated, and the patient also had no palpitations (Figure 2). We suspected that the switch caused the re-appearance of premature ventricular and atrial contractions rather than inducing the arrhythmia.We obtained informed consent from the patient. Our case report is the first case describing the effect of oral generic cibenzoline on premature contractions.

## Discussion

Atrial fibrillation is the most common cardiac arrhythmia in clinical practice. Atrial fibrillation can be treated with electrical cardioversion, catheter ablation, and pharmacological therapy. Pharmacological antiarrhythmic therapy can restore and maintain sinus rhythm; however, it may exert adverse effects such as ventricular arrhythmia and organ toxicity. Therefore, care must be taken to prevent such events.

Cibenzoline is a class I sodium channel blocker antiarrhythmic drug that can be used in limited countries. Cibenzoline also has a moderate calcium channel blocking (class IV) effect and potassium channel blocking (class III) effect [1]. It is used for the treatment of supraventricular and ventricular arrhythmias [2]. It is a commonly used drug in the treatment of atrial fibrillation. Cibenzoline is metabolized principally by CYP3A4 in the liver. We checked the patient's blood data to see if there were any indicators of disorders that induce arrhythmias, such as liver dysfunction, hyperkalemia, or hyperthyroidism. With drugs that have serious drug interaction potentials, such as amiodarone, changes in antiarrhythmic levels associated with formulation substitution may result in secondary changes in concentrations of other coadministered drugs, such as digoxin or warfarin [3]. In the present case, he had normal liver and renal functions; furthermore, he had taken no new drugs recently.

Generic drug use reduces costs to healthcare suppliers and consumers. The report of the Japanese Health Ministry showed that the diffusion rate of generic drugs increased to 79.2% as of April 2022. In the future, an increase in the generic drug diffusion rate is expected. Some reports have suggested that generic drugs may be less effective and safe than brand-name drugs [4]. One report linked the recurrence of atrial fibrillation with a rapid ventricular response to a quinidine formulation substitution [4]. On the other hand, in a study about propafenone, Amit et al. [1] reported that the generic formulation was found to be at least as safe and effective as the brand formulation with regard to atrial fibrillation recurrence, emergency room and hospital admissions, and necessity for concomitant therapy [5]. Another report evaluated the risk of thyroid dysfunction between patients using brand name versus generic formulations of amiodarone and found no difference in terms of incidence of thyroid dysfunction [6]. In general, however, there is little evidence of important clinical differences between generic and brand-name drugs in cardiovascular disease [7].

There are no case reports about the effect of generic cibenzoline. It is known that therapeutically equivalent generic drugs may differ in inactive ingredients and binder substances (Table 1). These factors may result in different degrees of absorption from the gastrointestinal tract in some patients [8]. We also need to consider the possibility that differences in blood levels of the 'active' ingredients may have caused differences in the effects of the drugs. This is because generic drugs are approved on the condition that the difference in blood concentration between the generic drug and the brand-name drug at the time of administration is within a certain range. In antiarrhythmic drugs, the therapeutic window may be narrower than other drugs. In this case, generic cibenzoline induced frequent premature ventricular and atrial contractions episodes.

**Table 1:** Differences in excipients between the brand (Cibenol®) and the generic (cibenzoline succinate) formulations. Bold indicates common excipients to generic and brand-name drugs.

Cibenol®	Cibenzoline Succinate
Crystalline cellulose, Carmellose calcium, Hydroxypropyl cellulose, Magnesium stearate, Polyvinyl acetal diethylamino acetate, Dimethylpolysiloxane, Carnauba wax	Crystalline cellulose, Carmellose calcium, Hypromellose, Magnesium stearate, Pregelatinized starch, Macrogol 6000, Talc, Titanium oxide, Carnauba wax

## Conclusions

In conclusion, clinicians should be aware of therapeutically equivalent and therapeutic windows of generic antiarrhythmic drugs.Therefore, at the time of the first administration, clinicians should treat generic drugs as if they are new drugs in terms of testing their safety. Awareness of possible effects when using generic antiarrhythmic drugs could increase the safety of pharmacological treatments for preventing cardiac arrhythmias.

## References

[1] Amit G, Rosen A, Wagshal AB (2004). Efficacy of substituting innovator propafenone for its generic formulation in patients with atrial fibrillation. Am J Cardiol.

[2] Chevalier P, Dacosta A, Chalvidan T, Bonnefoy E, Kirkorian G, Isaaz K, Touboul P (1998). Safety and tolerability of intravenous cibenzoline for acute termination of spontaneous sustained ventricular tachycardia: Cibenzoline and spontaneous VT. Int J Cardiol.

[3] Harron DW, Brogden RN, Faulds D, Fitton A (1992). Cibenzoline: a review of its pharmacological properties and therapeutic potential in arrhythmias. Drugs.

[4] Kesselheim AS, Misono AS, Lee JL, Stedman MR, Brookhart MA, Choudhry NK, Shrank WH (2008). Clinical equivalence of generic and brand-name drugs used in cardiovascular disease: a systematic review and meta-analysis. JAMA.

[5] Reiffel JA (2001). Issues in the use of generic antiarrhythmic drugs. Curr Opin Cardiol.

[6] Reiffel JA, Kowey PR (2000). Generic antiarrhythmics are not therapeutically equivalent for the treatment of tachyarrhythmias. Am J Cardiol.

[7] Tsadok MA, Jackevicius CA, Rahme E (2011). Amiodarone-induced thyroid dysfunction: brand-name versus generic formulations. CMAJ.

[8] Varley AB (1968). The generic inequivalence of drugs. JAMA.

